# The Incremental Induction of Neuroprotective Properties by Multiple Therapeutic Strategies for Primary and Secondary Neural Injury

**DOI:** 10.3390/ijms160819657

**Published:** 2015-08-19

**Authors:** Seunghoon Lee, Sookyoung Park, Jinyoung Won, Sang-Rae Lee, Kyu-Tae Chang, Yonggeun Hong

**Affiliations:** 1 Department of Physical Therapy, College of Biomedical Science & Engineering, Inje University, Gimhae 50834, Korea; E-Mail: stormyboy@nate.com; 2 Biohealth Products Research Center (BPRC), Inje University, Gimhae 50834, Korea; E-Mail: wy11167@naver.com; 3 Ubiquitous Healthcare & Anti-aging Research Center (u-HARC), Inje University, Gimhae 50834, Korea; E-Mail: charm-soo@hanmail.net; 4 Department of Physical Therapy, College of Life Sciences, Kyungnam University, Changwon 51767, Korea; 5 Department of Rehabilitation Science, Graduate School of Inje University, Gimhae 50834, Korea; 6 National Primate Research Center (NPRC), Korea Research Institute of Bioscience and Biotechnology (KRIBB), Ochang 28116, Korea; E-Mail: srlee@kribb.re.kr

**Keywords:** neural damages, risk factors, therapeutic intervention, melatonin, exercise

## Abstract

Neural diseases including injury by endogenous factors, traumatic brain injury, and degenerative neural injury are eventually due to reactive oxygen species (ROS). Thus ROS generation in neural tissues is a hallmark feature of numerous forms of neural diseases. Neural degeneration and the neural damage process is complex, involving a vast array of tissue structure, transcriptional/translational, electrochemical, metabolic, and functional events within the intact neighbors surrounding injured neural tissues. During aging, multiple changes involving physical, chemical, and biochemical processes occur from the molecular to the morphological levels in neural tissues. Among many recommended therapeutic candidates, melatonin also plays a role in protecting the nervous system from anti-inflammation and efficiently safeguards neuronal cells via antioxidants and other endogenous/exogenous beneficial factors. Therefore, given the wide range of mechanisms responsible for neuronal damage, multi-action drugs or therapies for the treatment of neural injury that make use of two or more agents and target several pathways may have greater efficacy in promoting functional recovery than a single therapy alone.

## 1. Risk Factors for Neural Injury

A substantial amount of evidence demonstrates that neural injury is associated with multiple pathological signaling pathways and that these mechanisms play a pivotal role in the development of disease processes and during the application of various therapies. This review will focus on neural injury induced by various sources including neurodegeneration, neurotrauma, and neuroinflammation.

Neurodegeneration is the progressive loss of the structure or function of neurons, including death [1,2]. It is well known that Parkinson’s disease (PD), Alzheimer’s disease (AD), Huntington’s disease (HD), and amyotrophic lateral sclerosis (ALS, or Lou Gehrig’s disease) occur as a result of neurodegenerative processes [3]. There is an increasingly enthusiastic interest in more fully understanding the molecular bases of the pathogenesis of these neurodegenerative diseases [3,4,5]. Although a variety of risk factors contribute to this type of disease, oxidative stress is a common element associated with neurodegenerative disease processes. Neuronal populations within the central nervous system (CNS) are especially susceptible to oxidative stress due to their limited capacity for regeneration, high metabolic rate, and iron/copper composition [6]. Mounting evidence indicates that, in the context of CNS injury, reactive oxygen species (ROS) and reactive nitrogen species (RNS) also contribute to protein aggregation and misfolding [7,8,9] and lead to damaging effects. An increase in oxidative stress via the action of dopamine may also lead to additional neuronal damage, mitochondrial injury, and altered cellular transport in PD [10]. Furthermore, oxidative damage may induce protein aggregates and neuronal dysfunction that can affect the activity of ion channels and pumps, neurotransmission, and axonal and dendritic transport in AD [11]. Sipione *et al.* [12]. demonstrated in animals that the presence of 3-nitropropionate, a mitochondrial toxin, results in neuropathology similar to that observed in human HD; this suggests that the pathogenesis of HD is also associated with oxidative stress and mitochondrial dysfunction. ALS is a devastating neurodegenerative disease and results in the degeneration of motor neurons, which leads to progressive muscle wasting, weakness, and paralysis and eventually culminates in respiratory failure and death [13]. Mutations in the Cu/Zn superoxide dismutase (*SOD*) gene are associated with familial ALS suggesting that ALS is also associated with oxidative damage [14].

Based on their etiology, most neural injuries are associated with several pathophysiological processes that progress from neurotrauma and/or neuroinflammation to neurodegeneration. Such neurotraumatic injuries include stroke (hemorrhage or ischemia), spinal cord injury (SCI), and traumatic brain injury (TBI) and are differentiated from chronic neurodegenerative CNS disorders such as AD, PD, HD, and ALS [6]. However, that it is still difficult to distinguish the biochemical pathways that are active during the “acute” and “chronic” phases of neural injury should be considered. Neurotraumatic injuries exhibit two major patho-anatomical zones based on the mechanism underlying the injury. The core zone has a lesion site, which results in necrotic neuronal death within minutes to hours following the insult [15], whereas the penumbra zone is the area surrounding a traumatic event such as hemorrhage, ischemia, contusion, or compression of neural tissue [16]. The neuroinflammatory response is also a key pathological mechanism underlying neural injury. Multiple sclerosis (MS), a chronic inflammatory and progressive disease of the CNS, eventually causes demyelination and axonal injury [17,18]. A large number of studies using an animal model of MS (experimental autoimmune encephalomyelitis; EAE) to investigate the pathogenesis of this autoimmune disease have found that oxidative stress plays a role in MS as well [19,20,21]. It has been proposed that an inflammatory response is related to classic neurodegeneration in diseases such as AD and PD [22,23]. One study using a mouse model of PD demonstrated that selective inhibitors of cyclooxygenase (COX)-2 exert neuroprotective effects following 1-methyl-4-phenyl-1,2,3,6-tertahydropyridine (MPTP)-induced neurotoxicity [24]. Nevertheless, one must use caution when defining the role of inflammatory responses in the pathogenesis of neural injury.

## 2. Window for Multi-Active Therapies: Primary and Secondary Injury

**Figure 1:**
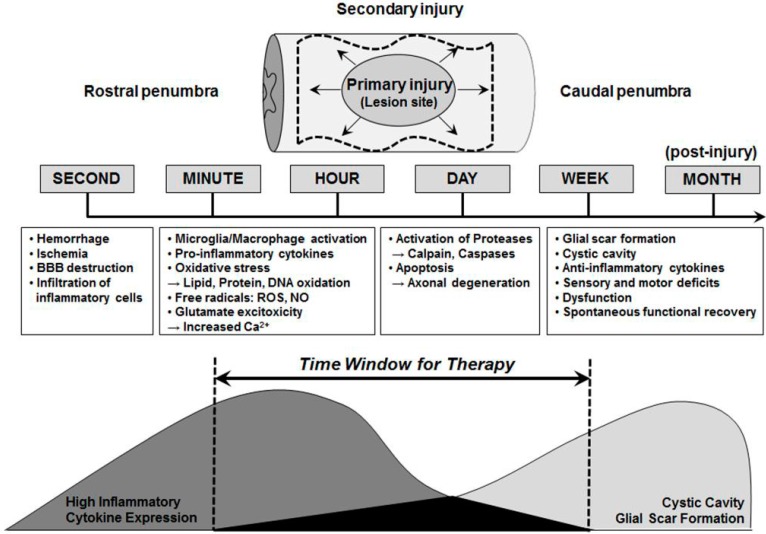
Time window for multi-active therapies. Time schedules of various events are progressed following neural injury including neurodegeneration, neurotrauma, and neuroinflammatory responses. These patholophysiological processes, which are distinguished as either primary or secondary injuries, possess considerable potential as therapeutic targets. This figure is adapted from Okano (2002) with some modifications [30].

Accumulating evidence demonstrates that multiple pathways are involved in the pathogenesis of neural injury and a number of mechanisms are activated following its onset (Figure 1). In particular, neurotraumatic injuries such as stroke, SCI, and TBI are characterized by two distinct pathophysiological mechanisms. Primary injury at the site of the lesion causes necrosis in neurons, which is likely to be irreversible, even with therapeutic intervention, within minutes to hours following injury [25]. In contrast, the secondary injury process is a prime target for therapeutic intervention because it involves a number of treatable mechanisms including the activation of microglia, infiltration of macrophages, secretion of pro-inflammatory cytokines (TNF-α, IFN-γ, *etc.*), intracellular calcium (Ca^2+^) influx, glutamate excitotoxicity, and inflammation [26]. Moreover, oxidative stress, which results in the presence of free radicals (ROS or nitric oxide (NO)), the oxidation of lipids, proteins, and DNA, and DNA damage, which leads to the activation of multiple cell death proteases (Calpains and Caspases) and the progressive apoptotic death of neurons and glia in the hours and weeks following the injury are involved [27,28,29]. The increase in lesion size over time following the injury is considered to be secondary injury damage, which spreads into the caudal penumbra.

Despite the use of various therapeutic interventions to attenuate the pathogenesis of neural injury, hormone therapies using estrogen (17β-estradiol; E_2_) and/or melatonin as neuroprotectants are thought to be effective approaches with several beneficial effects [31,32,33]. Both estrogen and melatonin are of particular interest for the treatment of neural injury because both of these compounds exert anti-inflammatory, antioxidant, anti-apoptotic, neurotrophic, and angiogenic actions *in vitro* and *in vivo* [34,35,36,37]. Estrogen has powerful anti-inflammatory effects via the attenuation of pro-inflammatory cytokine levels, intracellular Ca^2+^ influx, and glutamate excitotoxicity [38], while melatonin has strong antioxidative effects as a scavenger of free radicals such as ROS and NO [39]. Recently, it was shown that melatonin enhances the proliferation of neural stem cells (NSCs) [40], and that estrogen promotes their differentiation [41]. NSCs are multipotential progenitor cells that possess self-regeneration capabilities; thus, a single NSC is capable of creating diverse types of cells within the CNS including neurons, astrocytes, and oligodendrocytes [30]. Because of these characteristics, cell therapy with NSC transplantation has gained attention as a novel therapeutic intervention and, in fact, there is a growing interest in NSCs and neural progenitor cells in the fields of basic developmental biology and clinical therapy. It should be noted that these cell therapies must be applied within an optimal therapeutic time window.

## 3. Brain Aging and Neurodegeneration

Brain aging is characterized by progressive deficits in neurophysiological function that are frequently accompanied by age-related neurodegeneration [42]. It is widely recognized that the volume of the brain and/or the weight of the brain decreases with age at a rate of about 5% per decade after age 40 and possibly faster in individuals over the age of 70 [43]. It has been suggested that a decline in neuronal volume rather than in the number of cells contributes to changes in the aging brain. Additionally, there may be changes in synapses and dendritic arborization and spines [44]. Neuronal cell death via apoptosis is involved in all neurodegenerative diseases including AD, ischemic stroke, ALS, and PD. Therefore, a therapeutic agent that attenuates or prevents apoptotic cell death can be an effective strategy for the treatment of neurodegenerative diseases [45].

**Figure 2:**
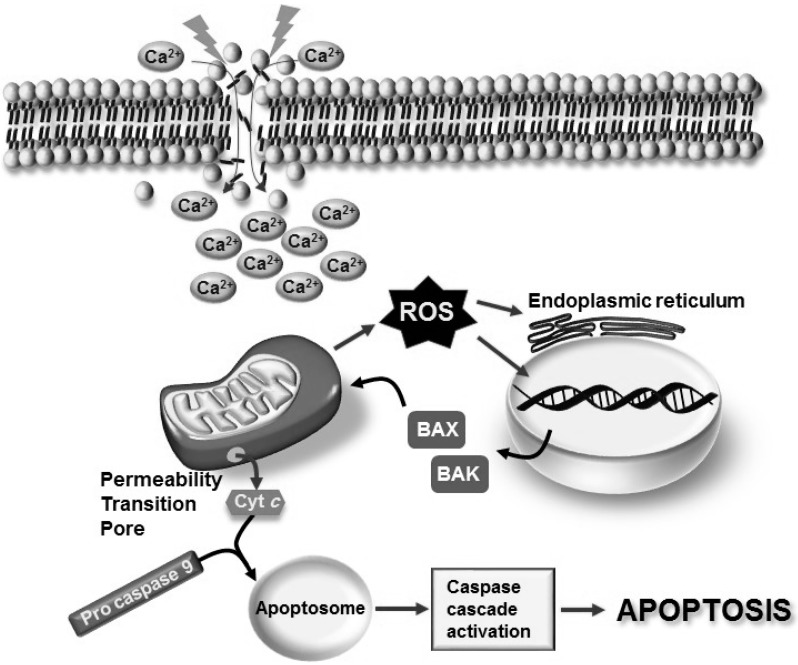
Apoptosis can result from the activation of two biochemical streams known as the extrinsic and intrinsic (mitochondrial) pathways. The intracellular apoptotic (mitochondrial) pathway is triggered by intracellular signals such as excessive Ca^2+^ influx and the over-generation of ROS from mitochondria. The initiator caspase 9 is activated and can catalyze the proteolytic maturation of executioner caspases, such as caspase 3, which mediate apoptosis. The aperture of the permeability transition pore is a point of no return in the mitochondrial pathway because it is associated with the activation of both caspase-dependent and caspase-independent mechanisms that eventually execute cell death. Cytochrome *c* is released into the cytosol and interacts with pro-caspase 9 to form the apoptosome. This results in the sequential activation of caspase 9 and executioner caspases, such as caspase 3, a process that is known as the caspase cascade. Finally, DNA damage could also modulate the induction of Bcl2 pro-apoptotic proteins, such as BAX and BAK, which favor the apoptotic process.

Several changes involving physical, chemical, and biological processes occur from the molecular to the morphological level under aging processes [44]. In particular, the alteration of mitochondrial function plays a key role in the neurodegenerative process of neuronal cell death via apoptotic signaling pathways [46]. There are two apoptotic signaling pathways: the extrinsic pathway via the activation of membrane Fas receptors and the intrinsic, or mitochondrial, pathway [46,47]. It is believed that the induction of apoptosis occurs by a sudden and/or sustained intracellular Ca^2+^ increase, which induces the activation of signals, such as ROS, which result in damage to DNA and endoplasmic reticulum (ER) stress [48]. In turn, this damage leads to the activation of the pro-apoptotic Bcl-2 homologues, Bcl-2-associated protein X (Bax), and Bcl-2-antagonist killer (Bak), which are members of the Bcl-2 family of proteins that tightly regulates the apoptotic process [46,47,49,50]. The mitochondrial pathway is a complex route because Bcl-2-regulated apoptosis requires Bax or Bak activation to cause mitochondrial damage. Members of the Bcl-2 family control the integrity of the mitochondrial membrane in healthy cells as well as its permeabilization, through oligomerization, in response to apoptotic stimuli and the formation of mitochondrial pores in the outer membrane. For this reason, specific mitochondrial pro-apoptotic proteins are produced in the cytoplasm [49,50,51]. A typical protein identified with pro-apoptotic functions is Cyt *c*, which is released from the mitochondria into the cytoplasm through the permeability transition pore (PTP). Several studies suggest that Cyt *c* triggers formation of the apoptosome, which triggers pro-caspase 9 and, in successive steps, caspase-mediated apoptosis via caspases 3, 6, and 7 [5] (Figure 2).

## 4. Role of Melatonin in Age-Related Neural Degeneration

Melatonin is released from the pinealocytes into the blood and cerebrospinal fluid in the third ventricle of the brain [52]. Over the last decade, melatonin has emerged as a very powerful free radical scavenger and antioxidant [53,54,55]. Produced endogenously or ingested as an exogenous supplement, melatonin is a potent indirect antioxidant via its stimulatory action on antioxidant enzymes [56,57,58,59] as well as a direct free radical scavenger [60]. It is well known that melatonin plays an important role in the attenuation of glutamate-mediated Ca^2+^ influx and inflammation by inhibiting levels of pro-inflammatory cytokines [61,62,63,64]. It has also been reported that melatonin is present in some subcellular compartments, such as the nucleus and mitochondria [65]. The occurrence of death signals involving oxidative stress indicates that mitochondria play a central role in apoptosis via the release of Cyt *c* and other apoptogenic factors [66,67,68]. Borner [68] reported that Bcl-2, Bcl-xL, and Bax, all members of the Bcl-2 family, regulate apoptosis through homodimerization and heterodimerization. While Bcl-2 is predominantly membrane-associated and localized in the nuclear envelope, ER, and mitochondrial membranes, a significant amount of Bcl-xL and most of the Bax proteins reside in the cytoplasmic fraction [69]. Andrabi *et al.* [70] showed that melatonin prevents the mitochondrial release of Cyt *c* in striatal neurons through direct inhibition of the mitochondrial PTP. In age-associated AD research, it has been suggested that melatonin supplementation improves circadian rhythmicity, produces beneficial effects on memory, and decreases agitated behavior, confusion, and “sundowning” [71]. Several studies have found that melatonin significantly ameliorates tau hyperphosphorylation and, more specifically, attenuates tau hyperphosphorylation induced by wortmanin [72], calyculin A [73,74,75], and okadaic acid [76] in N2a and SH-SY5Y neuroblastoma cells. Recently, Lin *et al.* [71] suggested that melatonin also plays a role in protecting the cholinergic system from anti-inflammation and efficiently safeguards neuronal cells from amyloid beta (Aβ)-mediated toxicity via antioxidant and anti-amyloid properties.

## 5. Endogenous Factors Induced by Forced Exercise

The brain is particularly sensitive to oxidative stress due to its high metabolic rate. It is well-established that oxidative stress is closely linked to the pathology of a variety of neurodegenerative diseases, including age-related disorders [77]. Due to the high reactivity of ROS, its short lifespan, and issues related to its direct detection, ROS levels are often judged based on alterations of antioxidant status or the accumulation of relatively stable products of lipid, protein, and DNA interactions. However, indicators of oxidative damage other than the concentration and reactivity of ROS are also influenced by the activity of repair systems [78,79]. Levels of the oxidative modification of lipids, proteins, and DNA are generally used as markers of oxidative damage and increase with age. However, other than the ROS-associated neurodegeneration that is associated with a significant ROS load, moderate amounts of these reactive species could have beneficial effects on signaling and neurogenesis [80]. Exercise is a potent modulator of certain neurotrophins and such agents are likely to be significantly involved in the beneficial effects of exercise on CNS function [55]. For example, although there is an increase in the generation of ROS during physical exercise, regular exercise is known to improve the physiological performance of skeletal and cardiac muscle and decrease the incidences of a wide range of diseases [81]. The positive systemic effects of exercise also include the brain, and it is clear that regular exercise is beneficial for brain function and could play an important prophylactic and therapeutic role in stroke, AD, and PD [78,82]. The effects of forced exercise are very complex and could include neurogenesis via the action of neurotrophic factors such as BDNF and NGF, increased angiogenesis, and decreased oxidative damage [83,84].

Previous studies have suggested that exercise may release antioxidant enzymes. Rats trained in swimming suffer from a significant augmentation of lipid peroxidation and increases in glutathione peroxidase (GPX) activity [85]. Moreover, the activities of antioxidant enzymes vary based on brain region and, accordingly, the effects of exercise are also dependent on brain region. In certain brain areas, such as the brain stem and corpus callosum, exercise results in the increased activity of SOD and GPX [85]. In stroke-prone spontaneously hypertensive rats, exercise training can inhibit sympathetic nerve activity by decreasing oxidative stress via a blockage of angiotensin II type 1 receptor A [86]. Neurogenesis-related research has suggested that regular treadmill training exerts a protective effect against sleep deprivation-induced spatial memory deficiencies via the induction of hippocampal signaling cascades that positively modulate basal and stimulated levels of key effectors such as P-CaMKII and BDNF and an attenuation of increases in the protein phosphatase calcineurin [87]. However, long-term periodic exercise training in rats does not appear to significantly alter lipid peroxidation levels in the brain [88]. Ogonovszky *et al.* [89] subjected rats to moderate exercise, very hard exercise, and over-training and observed beneficial effects on brain function and a reduced accumulation of ROS, even with very hard exercise and over-training.

It has been suggested that BDNF regulates brain development, neuroplasticity, neurogenesis, neurite outgrowth, synaptic plasticity, and cell survival [90]. Treadmill exercise has been shown to increase BDNF levels and enhance the activity of PKA/Akt/CREB and MAPK/CREB signaling pathways in the hippocampus of middle-aged and old rats [91]. Likewise, exercise and oxidative stress up-regulate the expression and protein content of BDNF [92] and exercise enhances the content of BDNF and TrkB, activates CREB, and increases the expression of BDNF to make neurons more resistant to oxidative stress, probably via an alteration of the redox state [79]. Other trophic factors enhanced by exercise include IGF-1 and VEGF. IGF-1 is essential for neurite growth, neurotransmitter synthesis and release, and is believed to be functionally associated with the action of BDNF [93]. Recent studies indicate that the exercise-mediated induction of VEGF levels is regulated by activation of the mammalian target of rapamycin (mTOR) pathway [94] and it is well established that exercise increases neurogenesis, a process which benefits brain function [90]. It has been suggested that BDNF is one of the major regulators of neurogenesis. VEGF is also heavily involved in neurogenesis and the effects of exercise in terms of VEGF content and mRNA expression seem to be dependent on the dose of exercise.

## References

[1] Rubinsztein D.C. (2006). The roles of intracellular protein-degradation pathways in neuro-degeneration. Nature.

[2] Bredesen D.E., Rao R.V., Mehlen P. (2006). Cell death in the nervous system. Nature.

[3] Bossy-Wetzel E., Schwarzenbacher R., Lipton S.A. (2004). Molecular pathways to neuro-degeneration. Nat. Med..

[4] Moore D.J., West A.B., Dawson V.L., Dawson T.M. (2005). Molecular pathophysiology of Parkinson’s disease. Annu. Rev. Neurosci..

[5] Niranjan R. (2013). Molecular Basis of etiological implications in Alzheimer’s Disease: Focus on neuroinflammation. Mol. Neurobiol..

[6] Smith J.A., Park S., Krause J.S., Banik N.L. (2013). Oxidative stress, DNA damage, and the telomeric complex as therapeutic targets in acute neurodegeneration. Neurochem. Int..

[7] Radak Z., Zhao Z., Goto S., Koltai E. (2011). Age-associated neurodegeneration and oxidative damage to lipids, proteins and DNA. Mol. Asp. Med..

[8] Rajdev S., Hara K., Kokubo Y., Mestril R., Dillmann W., Weinstein P.R., Sharp F.R. (2000). Mice overexpressing rat heat shock protein 70 are protected against cerebral infarction. Ann. Neurol..

[9] Hu B.R., Martone M.E., Jones Y.Z., Liu C.L. (2000). Protein aggregation after transient cerebral ischemia. J. Neurosci..

[10] Xu J., Kao S.Y., Lee F.J., Song W., Jin L.W., Yankner B.A. (2002). Dopamine-dependent neurotoxicity of α-synuclein: A mechanism for selective neurodegeneration in Parkinson disease. Nat. Med..

[11] Hirai K., Aliev G., Nunomura A., Fujioka H., Russell R.L., Atwood C.S., Johnson A.B., Kress Y., Vinters H.V., Tabaton M. (2001). Mitochondrial abnormalities in Alzheimer’s disease. J. Neurosci..

[12] Sipione S., Cattaneo E. (2001). Modeling Huntington’s disease in cells, flies, and mice. Mol. Neurobiol..

[13] D’Amico E., Factor-Litvak P., Santella R.M., Mitsumoto H. (2013). Clinical perspective on oxidative stress in sporadic amyotrophic lateral sclerosis. Free Radic. Biol. Med..

[14] Rosen D.R., Siddique T., Patterson D., Figlewicz D.A., Sapp P., Hentati A, Donaldson D., Goto J., O’Regan J.P., Deng H.X. (1993). Mutations in Cu/Zn superoxide dismutase gene are associated with familial amyotrophic lateral sclerosis. Nature.

[15] Turner R.C., Lucke-Wold B., Lucke-Wold N., Elliott A.S., Logsdon A.F., Rosen C.L., Huber J.D. (2013). Neuroprotection for ischemic stroke: moving past shortcomings and identifying promising directions. Int. J. Mol. Sci..

[16] Köhrmann M., Schellinger P.D., Schwab S. (2010). The only evidence based neuroprotective therapy for acute ischemic stroke: Thrombolysis. Best. Pract. Res. Clin. Anaesthesiol..

[17] Amedei A., Prisco D., D’Elios M.M. (2012). Multiple sclerosis: The role of cytokines in pathogenesis and in therapies. Int. J. Mol. Sci..

[18] Khademi M., Dring A.M., Gilthorpe J.D., Wuolikainen A., Al Nimer F., Harris R.A., Andersson M., Brundin L., Piehl F., Olsson T. (2013). Intense inflammation and nerve damage in early multiple sclerosis subsides at older age: A reflection by cerebrospinal fluid biomarkers. PLoS ONE.

[19] Park S., Nozaki K., Guyton M.K., Smith J.A., Ray S.K., Banik N.L. (2012). Calpain inhibition attenuated morphological and molecular changes in skeletal muscle of experimental allergic encephalomyelitis rats. J. Neurosci. Res..

[20] Shields D.C., Tyor W.R., Deibler G.E., Hogan E.L., Banik N.L. (1998). Increased calpain expression in activated glial and inflammatory cells in experimental allergic encephalomyelitis. Proc. Natl. Acad. Sci. USA..

[21] Honorat J.A., Kinoshita M., Okuno T., Takata K., Koda T., Tada S., Shirakura T., Fujimura H., Mochizuki H., Sakoda S. (2013). Xanthine oxidase mediates axonal and myelin loss in a murine model of multiple sclerosis. PLoS ONE.

[22] Meraz-Ríos M.A., Toral-Rios D., Franco-Bocanegra D., Villeda-Hernández J., Campos-Peña V. (2013). Inflammatory process in Alzheimer’s Disease. Front. Integr. Neurosci..

[23] More S.V., Kumar H., Kim I.S., Song S.Y., Choi D.K. (2013). Cellular and molecular mediators of neuroinflammation in the pathogenesis of Parkinson’s disease. Mediat. Inflamm..

[24] Gupta A., Kumar A., Kulkarni S.K. (2011). Targeting oxidative stress, mitochondrial dysfunction and neuroinflammatory signaling by selective cyclooxygenase (COX)-2 inhibitors mitigates MPTP-induced neurotoxicity in mice. Prog. Neuropsychopharmacol. Biol. Psychiatry.

[25] Beattie M.S., Farooqui A.A., Bresnahan J.C. (2000). Review of current evidence for apoptosis after spinal cord injury. J. Neurotrauma.

[26] Beattie M.S., Hermann G.E., Rogers R.C., Bresnahan J.C. (2002). Cell death in models of spinal cord injury. Prog. Brain Res..

[27] Pan J.Z., Ni L., Sodhi A., Aguanno A., Young W., Hart R.P. (2002). Cytokine activity contributes to induction of inflammatory cytokine mRNAs in spinal cord following contusion. J. Neurosci. Res..

[28] Sribnick E.A., Wingrave J.M., Matzelle D.D., Ray S.K., Banik N.L. (2003). Estrogen as a neuroprotective agent in the treatment of spinal cord injury. Ann. N. Y. Acad. Sci..

[29] Paterniti I., Esposito E., Mazzon E., Bramanti P., Cuzzocrea S. (2011). Evidence for the role of PI3-kinase-AKT-eNOS signalling pathway in secondary inflammatory process after spinal cord compression injury in mice. Eur. J. Neurosci..

[30] Okano H. (2002). Stem cell biology of the central nervous system. J. Neurosci. Res..

[31] Inagaki T., Kaneko N., Zukin R.S., Castillo P.E., Etgen A.M. (2012). Estradiol attenuates ischemia-induced death of hippocampal neurons and enhances synaptic transmission in aged, long-term hormone-deprived female rats. PLoS ONE.

[32] Park S., Lee S.K., Park K., Lee Y., Hong Y., Lee S., Jeon J.C., Kim J.H., Lee S.R., Chang K.T. (2012). Beneficial effects of endogenous and exogenous melatonin on neural reconstruction and functional recovery in an animal model of spinal cord injury. J. Pineal Res..

[33] Park K., Lee Y., Park S., Lee S., Hong Y., Lee S.K., Hong Y. (2010). Synergistic effect of melatonin on exercise-induced neuronal reconstruction and functional recovery in a spinal cord injury animal model. J. Pineal Res..

[34] Prokai-Tatrai K., Perjesi P., Rivera-Portalatin N.M., Simpkins J.W., Prokai L. (2008). Mechanistic investigations on the antioxidant action of a neuroprotective estrogen derivative. Steroids.

[35] Losordo D.W., Isner J.M. (2001). Estrogen and angiogenesis: A review. Arterioscler. Thromb. Vasc. Biol..

[36] Liu X.J., Yuan L., Yang D., Han W.N., Li Q.S., Yang W., Liu Q.S., Qi J.S. (2013). Melatonin protects against amyloid-β-induced impairments of hippocampal LTP and spatial learning in rats. Synapse.

[37] He H., Dong W., Huang F. (2010). Anti-amyloidogenic and anti-apoptotic role of melatonin in Alzheimer disease. Curr. Neuropharmacol..

[38] Pozzi S., Benedusi V., Maggi A., Vegeto E. (2006). Estrogen action in neuroprotection and brain inflammation. Ann. N. Y. Acad. Sci..

[39] Sheth D.S., Tajuddin N.F., Druse M.J. (2009). Antioxidant neuroprotection against ethanol-induced apoptosis in HN2-5 cells. Brain Res..

[40] Sotthibundhu A., Phansuwan-Pujito P., Govitrapong P. (2010). Melatonin increases proliferation of cultured neural stem cells obtained from adult mouse subventricular zone. J. Pineal Res..

[41] Sekiguchi H., Ii M., Jujo K., Thorne T., Ito A., Klyachko E., Hamada H., Kessler J.A., Tabata Y., Kawana M. (2013). Estradiol promotes neural stem cell differentiation into endothelial lineage and angiogenesis in injured peripheral nerve. Angiogenesis.

[42] Duan W. (2013). Sirtuins: From metabolic regulation to brain aging. Front. Aging Neurosci..

[43] Scahill R.I., Frost C., Jenkins R., Whitwell J.L., Rossor M.N., Fox N.C. (2003). A longitudinal study of brain volume changes in normal aging using serial registered magnetic resonance imaging. Arch. Neurol..

[44] Peters R. (2006). Ageing and the brain. Postgrad. Med. J..

[45] Camins A., Sureda F.X., Junyent F., Verdaguer E., Folch J., Beas-Zarate C., Pallas M. (2010). An overview of investigational antiapoptotic drugs with potential application for the treatment of neurodegenerative disorders. Expert Opin. Investig. Drugs.

[46] Galluzzi L., Blomgren K., Kroemer G. (2009). Mitochondrial membrane permeabilization in neuronal injury. Nat. Rev. Neurosci..

[47] Ribe E.M., Serrano-Saiz E., Akpan N., Troy C.M. (2008). Mechanisms of neuronal death in disease: Defining the models and the players. Biochem. J..

[48] Shah R.S., Lee H.G., Xiongwei Z., Perry G., Smith M.A., Castellani R.J. (2008). Current approaches in the treatment of Alzheimer’s disease. Biomed. Pharmacother..

[49] Kroemer G., Galluzzi L., Brenner C. (2007). Mitochondrial membrane permeabilization in cell death. Physiol. Rev..

[50] Brooks C., Wei Q., Feng L., Dong G., Tao Y., Mei L., Xie Z.J., Dong Z. (2007). Bak regulates mitochondrial morphology and pathology during apoptosis by interacting with mitofusins. Proc. Natl. Acad. Sci. USA.

[51] Caroppi P., Sinibaldi F., Fiorucci L., Santucci R. (2009). Apoptosis and human disease: Mitochondrion damage and lethal role of released cytochrome C as proapoptotic protein. Curr. Med. Chem..

[52] Skinner D.C., Malpaux B. (1999). High melatonin concentrations in third ventricular cerebrospinal fluid are not due to Galen vein blood recirculating through the choroid plexus. Endocrinology.

[53] Reiter R.J., Maestroni G.J.M. (1999). Melatonin in relation to the antioxidative defense and immune systems: Possible implications for cell and organ transplantation. J. Mol. Med..

[54] Reiter R.J., Tan D.X., Qi W.B., Manchester L.C., Karbownik M., Calvo J.R. (2000). Pharmacology and physiology of melatonin in the reduction of oxidative stress *in vivo*. Biol. Signals Recept..

[55] Hong Y., Palaksha K.J., Park K., Park S., Kim H.D., Reiter R.J., Chang K.T. (2010). Melatonin plus exercise-based neurorehabilitative therapy for spinal cord injury. J. Pineal Res..

[56] Tan D.X., Reiter R.J., Manchester L.C., Yan M.T., El-Sawi M., Sainz R.M., Mayo J.C., Kohen R., Allegra M., Hardeland R. (2002). Chemical and physical properties and potential mechanisms: Melatonin as a broad spectrum antioxidant and free radical scavenger. Curr. Top. Med. Chem..

[57] Reiter R.J., Tan D.X., Osuna C., Gitto E. (2000). Actions of melatonin in the reduction of oxidative stress: A review. J. Biomed. Sci..

[58] Reiter R.J., Tan D.X., Gitto E., Sainz R.M., Mayo J.C., Leon J., Manchester L.C., Vijayalaxmi, Kilic E., Kilic U. (2004). Pharmacological utility of melatonin in reducing oxidative cellular and molecular damage. Pol. J. Pharmacol..

[59] Rodriguez C., Mayo J.C., Sainz R.M., Antolín I., Herrera F., Martín V., Reiter R.J. (2004). Regulation of antioxidant enzymes: a significant role for melatonin. J. Pineal Res..

[60] Tan D.X., Chen L.D., Poeggeler B., Manchester L.C., Reiter R.J. (1993). Melatonin: A potent, endogenous hydroxyl radical scavenger. Endocr. J..

[61] Allegra M., Reiter R.J., Tan D.X., Gentile C., Tesoriere L., Livrea M.A. (2003). The chemistry of melatonins interaction with reactive species. J. Pineal Res..

[62] Cuzzocrea S., Reiter R.J. (2002). Pharmacological actions of melatonin in acute and chronic inflammation. Curr. Top. Med. Chem..

[63] Guerrero J.M., Reiter R.J. (2002). Melatonin-immune system relationships. Curr. Top. Med. Chem..

[64] Shinozuka K., Staples M., Borlongan C.V. (2013). Melatonin-based therapeutics for neuroprotection in stroke. Int. J. Mol. Sci..

[65] Menendez-Pelaez A., Reiter R.J. (1993). Distribution of melatonin in mammalian tissues: The relative importance of nuclear versus cytosolic localization. J. Pineal Res..

[66] Green D.R., Reed J.C. (1998). Mitochondria and apoptosis. Science.

[67] Kuwana T., Newmeyer D.D. (2003). Bcl-2 family proteins and the role of mitochondria in apoptosis. Curr. Opin. Cell Biol..

[68] Borner C. (2003). The Bcl-2 protein family: sensors and checkpoints for life-or-death decisions. Mol. Immunol..

[69] Juknat A.A., Méndez Mdel V., Quaglino A., Fameli C.I., Mena M., Kotler M.L. (2005). Melatonin prevents hydrogen peroxide-induced Bax expression in cultured rat astrocytes. J. Pineal Res..

[70] Andrabi S.A., Sayeed I., Siemen D., Wolf G., Horn T.F. (2004). Direct inhibition of the mitochondrial permeability transition pore: A possible mechanism responsible for anti-apoptotic effects of melatonin. FASEB J..

[71] Lin L., Huang Q.X., Yang S.S., Chu J., Wang J.Z., Tian Q. (2013). Melatonin in Alzheimer’s disease. Int. J. Mol. Sci..

[72] Deng Y.Q., Xu G.G., Duan P., Zhang Q., Wang J.Z. (2005). Effects of melatonin on wortmannin-induced tau hyperphosphorylation. Acta Pharmacol. Sin..

[73] Li X.C., Wang Z.F., Zhang J.X., Wang Q., Wang J.Z. (2005). Effect of melatonin on calyculin A-induced tau hyperphosphorylation. Eur. J. Pharmacol..

[74] Li S.P., Deng Y.Q., Wang X.C., Wang Y.P., Wang J.Z. (2004). Melatonin protects SH-SY5Y neuroblastoma cells from calyculin A-induced neurofilament impairment and neurotoxicity. J. Pineal Res..

[75] Yang X., Yang Y., Fu Z., Li Y., Feng J., Luo J., Zhang Q., Wang Q., Tian Q. (2011). Melatonin ameliorates Alzheimer-like pathological changes and spatial memory retention impairment induced by calyculin A. J. Psychopharmacol..

[76] Wang Y.P., Li X.T., Liu S.J., Zhou X.W., Wang X.C., Wang J.Z. (2004). Melatonin ameliorated okadaic-acid induced Alzheimer-like lesions. Acta Pharmacol. Sin..

[77] Radák Z., Ihasz F., Koltai E., Goto S., Taylor A.W., Boldogh I. (2014). The redox-associated adaptive response of brain to physical exercise. Free Radic. Res..

[78] Radák Z., Hart N., Sarga L., Koltai E., Atalay M., Ohno H., Boldogh I. (2010). Exercise plays a preventive role against Alzheimer’s disease. J. Alzheimers Dis..

[79] Rothman S.M., Mattson M.P. (2013). Activity-dependent, stress-responsive BDNF signaling and the quest for optimal brain health and resilience throughout the lifespan. Neuroscience.

[80] Radák Z., Zhao Z., Koltai E., Ohno H., Atalay M. (2013). Oxygen consumption and usage during physical exercise: the balance between oxidative stress and ROS-dependent adaptive signaling. Antioxid. Redox Signal..

[81] Radák Z., Chung H.Y., Goto S. (2005). Exercise and hormesis: oxidative stress-related adaptation for successful aging. Biogerontology.

[82] Mattson M.P., Magnus T. (2006). Ageing and neuronal vulnerability. Nat. Rev. Neurosci..

[83] Cotman C.W., Berchtold N.C. (2002). Exercise: A behavioral intervention to enhance brain health and plasticity. Trends. Neurosci..

[84] Cotman C.W., Engesser-Cesar C. (2002). Exercise enhances and protects brain function. Exerc. Sport Sci. Rev..

[85] Hara M., Iigo M., Ohtani-Kaneko R., Nakamura N., Suzuki T., Reiter R.J., Hirata K. (1997). Administration of melatonin and related indoles prevents exercise-induced cellular oxidative changes in rats. Biol. Signals.

[86] Kishi T., Hirooka Y., Katsuki M., Ogawa K., Shinohara K., Isegawa K., Sunagawa K. (2012). Exercise training causes sympathoinhibition through antioxidant effect in the rostral ventrolateral medulla of hypertensive rats. Clin. Exp. Hypertens..

[87] Zaggar M., Dao A., Alhaider I., Alkadhi K. (2013). Regular treadmill exercise prevents sleep deprivation-induced disruption of synaptic plasticity and associated signaling cascade in the dentate gyrus. Mol. Cell. Neurosci..

[88] Radak Z., Kaneko T., Tahara S., Nakamoto H., Pucsok J., Sasvari M., Nyakas C., Goto S. (2001). Regular exercise improves cognitive function and decreases oxidative damage in rat brain. Neurochem. Int..

[89] Ogonovszky H., Berkes I., Kumagai S., Kaneko T., Tahara S., Goto S., Radák Z. (2005). The effects of moderate-, strenuous- and over-training on oxidative stress markers, DNA repair, and memory, in rat brain. Neurochem. Int..

[90] Van Praag H., Kempermann G., Gage F.H. (1999). Running increases cell proliferation and neurogenesis in the adult mouse dentate gyrus. Nat. Neurosci..

[91] Marosi K., Felszeghy K., Mehra R.D., Radak Z., Nyakas C. (2012). Are the neuroprotective effects of estradiol and physical exercise comparable during ageing in female rats?. Biogerontology.

[92] Mattson M.P., Maudsley S., Martin B. (2004). A neural signaling triumvirate that influences ageing and age-related disease: Insulin/IGF-1, BDNF and serotonin. Ageing Res. Rev..

[93] Anlar B., Sullivan K.A., Feldman E.L. (1999). Insulin-like growth factor-1 and central nervous system development. Horm. Metab. Res..

[94] Elfving B., Christensen T., Ratner C., Wienecke J., Klein A.B. (2013). Transient activation of mTOR following forced treadmill exercise in rats. Synapse.

